# Small extracellular vesicles derived from human MSCs prevent allergic airway inflammation via immunomodulation on pulmonary macrophages

**DOI:** 10.1038/s41419-020-2606-x

**Published:** 2020-06-01

**Authors:** Shu-Bin Fang, Hong-Yu Zhang, Xiang-Ci Meng, Cong Wang, Bi-Xin He, Ya-Qi Peng, Zhi-Bin Xu, Xing-Liang Fan, Zhang-Jin Wu, Zi-Cong Wu, Song-Guo Zheng, Qing-Ling Fu

**Affiliations:** 1 Otorhinolaryngology Hospital, The First Affiliated Hospital, Sun Yat-sen University, 58 Zhongshan Road II, Guangzhou, China; 2 Department of Internal Medicine, Ohio State University College of Medicine and Wexner Medical Center, Columbus, OH United States

**Keywords:** Mesenchymal stem cells, Asthma

## Abstract

Allergic airway inflammation is a major public health disease that affects up to 300 million people in the world. However, its management remains largely unsatisfactory. The dysfunction of pulmonary macrophages contributes greatly to the development of allergic airway inflammation. It has been reported that small extracellular vesicles derived from mesenchymal stromal cells (MSC-sEV) were able to display extensive therapeutic effects in some immune diseases. This study aimed to investigate the effects of MSC-sEV on allergic airway inflammation, and the role of macrophages involved in it. We successfully isolated MSC-sEV by using anion exchange chromatography, which were morphologically intact and positive for the specific EV markers. MSC-sEV significantly reduced infiltration of inflammatory cells and number of epithelial goblet cells in lung tissues of mice with allergic airway inflammation. Levels of inflammatory cells and cytokines in bronchoalveolar lavage fluid were also significantly decreased. Importantly, levels of monocytes-derived alveolar macrophages and M2 macrophages were significantly reduced by MSC-sEV. MSC-sEV were excreted through spleen and liver at 24 h post-administration in mice, and were able to be taken in by macrophages both in vivo and in vitro. In addition, proteomics analysis of MSC-sEV revealed that the indicated three types of MSC-sEV contained different quantities of proteins and shared 312 common proteins, which may be involved in the therapeutic effects of MSC-sEV. In total, our study demonstrated that MSC-sEV isolated by anion exchange chromatography were able to ameliorate Th2-dominant allergic airway inflammation through immunoregulation on pulmonary macrophages, suggesting that MSC-sEV were promising alternative therapy for allergic airway inflammation in the future.

## Introduction

Allergic airway inflammation is a chronic airway disease characterized by peribronchial inflammation, reversible airway obstruction, and airway hyper responsiveness^1^. Several immune-cells including T cells, dendritic cells, epithelial cells, group II innate lymphoid cells (ILC2s), and macrophages were involved in the pathology of asthma. We and others have previously reported that mesenchymal stromal cells (MSCs) exhibited unique immunomodulatory properties on the regulation of macrophages^2,3^, dendritic cells^4^, and T cells^5^, and inhibited both eosinophilic and neutrophilic asthmatic inflammation^6,7^. It was reported that the immunomodulation of MSCs are mainly attributed to the cell–cell contact, paracrine functions, and releasing of exosomes^5,8,9^.

Exosomes are extracellular vesicles released through exocytosis of multivesicular bodies, which range from 30–150 nm in diameter. They carry a complex cargo of proteins, nucleic acids, and lipids, and are proposed as novel carriers in intercellular communication^10^. Since exosomes isolated by currently available methods are often a mixed population of extracellular vesicles (EV), in this study we termed “exosomes” as “small extracellular vesicles (sEV)” as indicated in the Minimal Information for Studies of Extracellular Vesicles 2018 (MISEV 2018)^11^. MSC-sEV distinguish from MSCs in that they are more shelf-stable, biologically safer, and less immunogenic. Increasing studies have demonstrated that sEV derived from MSCs elicited similar therapeutic effects to their parent cells in some intractable diseases^9,12^. Recently, we reported that MSC-sEV alleviated ILC2-dominant mouse allergic airway inflammation^13^, and some other studies have also reported that MSC-sEV were effective in allergic airway inflammation of mouse^14,15^. However, the mechanisms with regards to how MSC-sEV regulate allergic asthma remain largely unknown, and need to be further elucidated.

Macrophages are initial innate immunocytes that display diverse function in response to various environmental stimuli, which strikingly promotes the development and sustainability of innate and adaptive immune responses^16^. New investigations into the pathogenesis of severe asthma indicated that pulmonary macrophages played an important role in the development of allergic airway inflammation^17^. In lung tissues, alveolar macrophages (AMs) that reside in the surface of alveolar lumen are able to display diverse function when being continuously exposed to various environmental stimuli^18^. Although tissue-resident AMs (TR-AMs) mainly exhibit protective effects during homeostasis^19^, they could be polarized towards M1 or M2 phenotypes in response to inhaled pathogens and allergens, and thus contribute to the development of allergic airway inflammation^20–23^. In addition, peripheral monocytes could be rapidly recruited to the lung tissues and differentiate to macrophages, namely monocytes-derived alveolar macrophages (Mo-AMs) during the inflammatory process, which are further polarized towards proinflammatory phenotypes that play an important role in the development of asthma^24^. All these evidences suggested that the aberrant differentiation and dysfunction of macrophages significantly contributed to the pathogenesis of asthma. It has been previously reported that MSC-sEV elicited therapeutic effects through immunoregulation on pulmonary macrophages in some lung diseases, such as bronchopulmonary dysplasia^25^, pulmonary hypertension^26^, and lung injury^27^. Therefore, we hypothesized that MSC-sEV could possibly ameliorate allergic airway inflammation through regulation on the differentiation and polarization of pulmonary macrophages.

Currently, differential ultracentrifugation is the most widely used method for isolation of sEV^28^. However, it has been well acknowledged that this method was not applicable for mass production of MSC-sEV. In recent years, it is reported that anion exchange chromatography was one of the most promising isolation methods for separation of MSC-sEV^29^. Of note, anion exchange chromatography is efficient and scalable in the isolation of high-purity MSC-sEV. Thus, we modified this method for purification of sEV released by MSCs derived from induced pluripotent stem cells (iPSC-MSCs), which were phenotypically and functionally analogous to adult MSCs and were more proliferative as well^5,30^.

In this study, we sought to evaluate the therapeutic effects of MSC-sEV on allergic airway inflammation, and investigate the effects of MSC-sEV on macrophages in the setting of allergic airway inflammation.

## Methods and materials

### Animal

Female BALB/c mice aged 4–6 weeks were purchased from Beijing Vital River Laboratory Animal Technology Co., Ltd., and maintained in specific pathogen-free Animal Experimental Center of North Campus, Sun Yat-sen University. The protocols for animal experiments were reviewed and approved by the Ethics Committee of Sun Yat-sen University.

### Collection of sEV-enriched MSC supernatants

Human iPSC-MSCs used in this study were generated and identified as we reported previously^4^. Briefly, iPSC-MSCs were expanded to passage 8 and cryopreserved at 2 × 10^6^ cells per vial to create a cell bank to minimize the variation among different preparation of sEV. For each preparation, one vial of iPSC-MSCs was recovered in two 150-cm^2^ tissue culture plates (Corning, Oneonta, NY, USA) and incubated in complete culture medium (CCM) for 2–3 days. The cells were then seeded in twenty 150-cm^2^ tissue culture plates, and incubated in CCM for another 3–4 days. After the cells reached about 80% confluency, the CCM were replaced with chemically defined and protein free (CDPF) medium^29^. Then CDPF medium were discarded 6 h later and the cells were incubated with fresh CDPF medium for another 42 h. The medium recovered between 6 and 48 h in total volume of about 600 mL were finally used for isolation of sEV. Bone marrow-derived MSCs (BM-MSCs) were purchased commercially from Cyagen Biosciences (https://apac.cyagen.com/, CAT# HUXMA-01001). Gingiva-derived MSCs (G-MSCs) were prepared as reported in our previous study^31^. Similarly, BM-MSCs and G-MSCs were cultured in CCM (MEMα medium supplemented with 16.7% FBS, 100 U/mL penicillin/streptomycin for BM-MSCs, MEMα medium supplemented with 10% FBS, 100 U/mL penicillin/streptomycin for G-MSCs), and then incubated with CDPF for sEV separation.

### Isolation of MSC-sEV

MSC-sEV were isolated by applying a scalable protocol of anion exchange chromatography as reported previously with some modifications^29^. Briefly, Econo-Pac columns (Bio-Rad Laboratories, Hercules, CA, USA) were each packed with anion exchange resin (Q Sepharose Fast Flow; GE Health Care Life Science, Pittsburgh, PA, USA) and equilibrated with Equilibration Buffer. Next, the supernatant was loaded into each column and the proteomic impurities were removed by applying Wash Buffer. Then, MSC-sEV were eluted from the column by loading Elution Buffer continuously for eight times. Finally, the concentration of MSC-sEV in each fraction was determined by Bradford protein assay and ELISA for CD63. The peak fractions were collected for phosphate buffered saline (PBS) dialysis. The final concentration of MSC-sEV was determined by Bradford protein assay and nanoparticle tracking analysis (Nanosight NS300; Malvern, UK). The isolated MSC-sEV were either used for study immediately or stored at −80 °C for preservation. To prevent endotoxin contamination of MSC-sEV, all the buffers and materials used for MSC-sEV isolation were endotoxin-free. The equilibration buffer contained 50 mM sodium phosphate buffer and 50 mM sodium chloride. The wash buffer contained 50 mM sodium phosphate buffer and 100 mM sodium chloride. The elution buffer contained 50 mM sodium phosphate buffer and 500 mM sodium chloride.

### Transmission electron microscopy (TEM)

Fresh MSC-sEV samples were fixed with 2% glutaraldehyde (Sigma, Saint Louis, MO, USA) for 30 min. Next, 10 µL of fixed samples were incubated with copper grids with carbon-coated formvar film for 10 min. Then, the grads were incubated with 30 µL of 3% uranyl acetate (Sigma, Saint Louis, MO, USA, pH = 7.0) for 5 min after three washes with H_2_O. The images of MSC-sEV were photographed using a TEM instrument (H7650; HITACHI, Tokyo, Japan). All the procedures mentioned above were performed and technically supported by Guangdong Institute of Microbiology.

### SDS -PAGE and LC-MS/MS analysis of MSC-sEV

The MSC-sEV were separated with 10% sodium dodecyl sulfate polyacrylamide gel electrophoresis (SDS-PAGE) and stained with Coomassie blue. The stained gel bands were manually cut into equal size, detained and suggested to in-gel trypsin digestion. The tryptic peptides were analyzed using nano liquid chromatography-tandem mass spectrometry (LC-MS/MS) (Shimadzu Co., Japan) coupled to a Q-Exactive HF (Thermo Fisher Scientific, San Jose, CA). Briefly, the digested samples were desalted and separated in a column (75 μm × 15 cm) equipped with resins. The eluted peptides were then electrosprayed into nanoESI source at a voltage of 1.6 kV. All MS/MS spectra were acquired by data-dependent acquisition.

### Transfection of MSCs with mCherry-CD63

The lentivirus (LV) vectors encoding with mCherry-CD63 fusion gene driven by the EF1A promoter (pLV [Exp]-Puro-EF1A > mCherry (ns):hCD63, Supplementary Fig. 1a) was generated in Cyagen Biosciences Inc. (Guangzhou, Guangdong, CN). Human iPSC-MSCs were seeded in a 6-well plate at 2 × 10^5^ cells/well and the cells were incubated with 2 × 10^7^ lentivirus vectors per well for 18 h. The supernatants were then removed and the cells were further incubated with fresh CCM for another 48 h. The transfected iPSC-MSCs were characterized by means of fluorescence microscope and flow-cytometry analysis, which both demonstrated that more than 90% of iPSC-MSCs were successfully transduced (Supplementary Fig. 1b and 1c). The cells were further used for isolation of iPSC-MSC-sEV and about 90% of the sEV were labeled with mCherry as shown by flow-cytometry analysis (Supplementary Fig. 1d).

### Generation and stimulation of human and mouse macrophages

To generate human macrophages, human buffy coats, provided by Guangzhou Blood Center, were used for isolation of peripheral blood mononuclear cells (PBMCs) by Ficoll–Paque premium density gradient centrifugation (1.078 g/mL, GE Healthare, England, UK). Exemption of written informed consent for blood buffy coats was approved by the Ethics Committee of The First Affiliated Hospital, Sun Yat-sen University, China. Then CD14^+^ monocytes were isolated from PBMCs by using MSCS CD14 MicroBeads (MiltenyiBiotec, Bergisch Gladbach, Germany) according to the manufacturer’s instructions. The CD14^+^ monocytes were seeded in 24-well plate at 5 × 10^5^ cells/well, and differentiated into mature macrophages in RPMI 1640 medium supplemented with 10% FBS (Gibco, Carlsbad, CA, USA), penicillin/streptomycin (Gibco, Carlsbad, CA, USA), and 20 ng/mL recombinant murine macrophage colony-stimulating factor (M-CSF; Peprotech, Rocky Hill, NJ, USA) for 7 days. The macrophages were characterized by means of flow-cytometry analysis and white light microscopy (Supplementary Fig. 2).

For isolation of mouse peritoneal macrophages, BALB/c mice were euthanized by cervical dislocation and sterilized with 75% ethanol, and peritoneal cells were harvested from the peritoneal cavity of the mice by lavage with 5 mL PBS with 2 mM EDTA·2Na. Then macrophages were further purified from the peritoneal cells by plastic adherence method. Peritoneal macrophages were cultured in RPMI 1640 supplemented with 10% FBS and penicillin/streptomycin. Additionally, in some experiments, Raw 264.7 macrophages (ATCC, Manassas, VA, USA. https://www.atcc.org/products/all/TIB-71.aspx), were used for evaluation of the immunoregulatory effects of MSC-sEV.

To determine the effects of MSC-sEV on the polarization of M2 macrophages, human, mouse peritoneal or Raw 264.7 macrophages were pretreated with 50 ng/mL IL-4 (R&D System, Minneapolis, MN, USA) for 24 h, and then incubated for another 24 h in the presence or absence of 1 × 10^9^/mL MSC-sEV. To evaluate the effects of MSC-sEV on the maturation of human macrophages, human monocytes were differentiated into macrophages in the presence of 1 × 10^9^/mL MSC-sEV. To examine the effects of MSC-sEV on the viability of macrophages, mouse macrophages were treated with or without 1 × 10^9^/mL MSC-sEV for 24 h. The cells were finally collected for PCR analysis or flow-cytometry analysis as indicated.

### The uptake of human iPSC-MSC-sEV by macrophages

For in vitro uptake experiments, human macrophages were plated onto round glass coverslips in 24-well plate at 3 × 10^5^ cells/well and incubated in 0.5 mL RPMI 1640 medium supplemented with 10% FBS and penicillin/streptomycin for 24 h. Then 5 × 10^8^ mCherry-labeled iPSC-MSC-sEV were added to each well, and incubated with macrophages for indicated time points. Then macrophages on the coverslips were further stained with 4′,6-Diamidine-2′-phenylindole dihydrochloride (DAPI) (Molecular Probes, Eugene, OR, USA) and photographed using laser scanning confocal microscope (LSM 780; Zeiss, Jena, Germany).

For in vivo uptake experiments, the mice were administered with 1.5 × 10^10^ mCherry-labeled iPSC-MSC-sEV and sacrificed at indicated time points. Total lung cells were isolated and mean fluorescence index (MFI) of mCherry in macrophages were analyzed by means of flow cytometry.

### Mouse model of allergic asthma

An ovalbumin (OVA)-induced eosinophilic asthma mouse model was developed as we previously described with some modifications^7^. Briefly, mice were sensitized intraperitoneally with 40 µg OVA (Sigma, St. Louis, MO, USA) and 4 mg aluminum /magnesium hydroxide (Thermo Scientific, Rockford, IL, USA) on days 0, 7, and 14 and then challenged with 5% OVA aerosols on days 21–23. The mice were treated with 1.5 × 10^10^ iPSC-MSC-sEV (OVA/OVA/iPSC-MSC-sEV, *N* = 5) or the same volume of PBS (OVA/OVA/PBS, *N* = 5) per day on day 20 and 2 h before challenge on day 21 and day 22. Control mice were treated with the same amount of PBS accordingly during the phases of sensitization, challenge and treatment (PBS/PBS/PBS, *N* = 5). All of the mice were finally sacrificed at 4 h after the last challenge. Bronchoalveolar lavage fluids (BALF) were harvested by three consecutive lavages with ice-cold PBS and centrifuged at 300 × g for 5 min. The supernatants were used for detection of cytokines by ELISA, and the pellets were used for analysis of inflammatory cells by flow cytometry. Lung tissues were used for pathologic analyses of pulmonary inflammation and quantitative real-time PCR (qRT-PCR) for macrophage-specific markers.

### In vivo imaging of MSC-sEV

Mice were administered with 3 × 10^10^ mCherry-labeled iPSC-MSC-sEV at the indicated time points. After the mice were sedated with isoflurane inhalation anesthesia, whole-body imaging of the living mice was performed to detect the distribution of mCherry-labeled iPSC-MSC-sEV on an in vivo xtreme imaging system (Bruker, Billerica, MA, USA). Then, the lungs, hearts, livers, kidneys, and spleens of the mice were surgically exposed and collected for further detection of the fluorescence intensity of mCherry. All the procedures of the imaging were commercially supported by Guangzhou RiboBio Co., Ltd.

### Flow-cytometry analysis

For the characterization of surface EV markers, 100 µL of iPSC-MSC-sEV were incubated with 20 µL of 4-µm-diameter anti-CD63-coated magnetic beads (Life Technologies, AS, Norway) overnight to coat sEV onto the beads as described previously^13^. The sEV-coated beads were stained with anti-CD9-PE, anti-CD63-FITC and anti-CD81-APC at RT for 30 min and washed three times with 500 µL of isolation buffer (PBS containing 0.1% bovine serum albumin) for further analysis. For in vivo detection of iPSC-MSC-sEV uptaken by macrophages, lung tissues were minced and incubated in D-Hank’s buffer containing 1 mg/mL collagenase type IA (Life Technologies, Carlsbad, CA, USA) and 50 μg/mL DNase I (Sigma, St. Louis, MO, USA) at 37 °C for 1 h. The digested lung tissues were minced against 70-µm strainers (Fisher Scientific, Pittsburgh, PA, USA) to obtain a single-cell suspension, which were stained with antibodies to CD45-APC-A700 and F4/80-FITC for analysis of pulmonary macrophages. For analysis of different inflammatory cells in mouse BALF, pellets were stained with antibodies to CD45-FITC, CD11b-APC-Cy7, CD64-PE, Ly-6G-Alexa 700, Siglec-F-Alexa 647, CD86-APC, and CD206-BV605 following the gating strategies as reported previously (Supplementary Fig. 3a)^32^. For studying the effects of MSC-sEV on the maturation of human macrophages cultured in vitro, the cells were first stained with antibodies to cell surface markers of CD16-PE and CD206-PE-Cy7 directly, or intracellular marker of CD68-PE-Cy7 after fixation and permeabilization. For studying the effects of MSC-sEV on polarization of human macrophages, the cells were stained with CD86-PE and CD206-PE-Cy7. For studying the effects of MSC-sEV on the viability of macrophages, the rate of apoptotic peritoneal macrophages was analyzed using apoptosis flow cytometry kit (BD Biosciences, San Jose, CA, USA). All the flow-cytometry antibodies were commercially purchased from BD Biosciences, San Jose, CA, USA. All the samples were detected on a CytoFLEX Flow Cytometer (Beckman Coulter, Hercules, CA, USA) and analyzed using CytExpert software (Beckman Coulter, Hercules, CA, USA).

### Enzyme-linked immunosorbent assay (ELISA)

Levels of cytokines in the mouse BALF and in vitro cell culture supernatants were assessed by using ELISA kit (R&D Systems, Minneapolis, MN, USA) following manufacturer’s instructions. Results were read with a BioTek Gen 5 microplate reader (BioTek, Winooski, VT, USA).

### Extraction of RNA and qRT-PCR analysis

Total RNA was extracted from mouse lung tissues and peritoneal macrophages or Raw 264.7 macrophages in in vitro experiments using RNAiso Plus reagent (Takara Bio Inc., Japan), Complementary DNA (cDNA) was synthesized with PrimeScript RT Master Mix (Takara Bio Inc., Japan) on a Veriti Thermal Cycler (Applied Biosystems, Foster, CA, USA). Quantitative real-time PCR was performed using SYBR Premix ExTaq (Takara Bio Inc., Japan) on an ABI 7500 Real Time PCR Systerm (Applied Biosystems, Foster, CA, USA). Data were calculated by 2^−ΔΔCt^ method and levels mRNA were normalized to GADPH. The primers used in our study were shown in Supplementary Table 1.

### Western blot

The concentrations of MSC-sEV samples and MSC lysates were determined by Pierce™ BCA protein assay (Thermo Fisher Scientific, Rockford, IL, USA) and denatured in SDS loading buffer at 95 °C for 5 min. Briefly, 10 µg of the cell lysates and MSC-sEV were loaded and separated by 10% SDS-PAGE. The proteins were transferred to polyvinylidene difluoride membranes (Roche Diagnostics, Mannheim, Germany) at 15 V for 1 h and blocked with 5% skim milk. Subsequently, the membrane was incubated with rabbit antibodies to CD9 (1:2000), CD63 (1:2000), CD81 (1:2000), Alix (1:5000), tumor susceptibility gene (TSG101, 1:1000), and Calnexin (1:1000) overnight at 4 °C. After three washes with Tris Buffered Saline with Tween 20 (TBST) buffer, the membranes were incubated with HRP conjugated anti-rabbit IgG for 1 h at RT. The membranes were then washed, incubated with Enhanced Chemiluminescence Plus (Millipore Corporation, Billerica, MA, USA) and photographed using an imaging analysis system (ImageQuant LAS 4000, GE Health Care Life Science, Uppsala, Sweden). All the primary antibodies were purchased from Abcam, Cambridge, UK and the secondary antibody was purchased from Jackon ImmunoResearch, West Grove, PA, USA.

### Statistical analysis

Statistical analyses were performed using Prism software (GraphPad Software, La Jolla, CA, USA). Statistical significance was assessed by using unpaired or paired independent 2-tailed Student’s *t*-test for single comparisons or one-way analysis of variance (ANOVA) with Tukey’s correction for multiple comparisons as indicated. Similar variance was found between the groups that are being statistically compared. All values were expressed as mean ± SEM for each group. A *P-*value less than 0.05 was considered statistically significant.

## Results

### Isolation and characterization of MSC-sEV

**Fig. 1:**
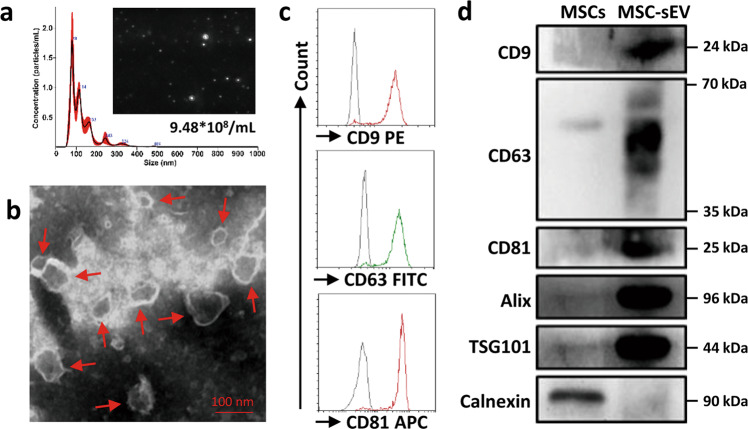
Characterization of iPSC-MSC-sEV. **a** Nanoparticle tracking analysis of iPSC-MSC-sEV with 1:100 dilution. **b** Transmission electron microscopy for iPSC-MSC-sEV. **c** Flow-cytometry analysis of exosomal surface markers on iPSC-MSC-sEV. **d** Western blot analysis of exosomal specific markers in iPSC-MSC-sEV. MSCs mesenchymal stromal cells, sEV small extracellular vesicles. Scale bar 100 nm.

In order to minimize variations among different preparations of MSC-sEV, iPSC-MSCs with homogeneous efficacy were stored in a cell bank. For preparation of sEV-enriched medium, iPSC-MSCs were cultured with CCM in twenty 150 cm^2^ cell culture plates and the CCM were replaced with CDPF after the cell reach 70–80% confluency. MSC-sEV were then isolated with the laboratory-scale anion exchange chromatography, which is extremely promising in that it could be scaled up to industrial production for clinical application in the future. The isolated MSC-sEV were in a range of 50–150 nm in diameter and had a concentration of approximately 1 × 10^11^/mL as determined by nanoparticle tracking analysis (Fig. 1a). The protein level of MSC-sEV was about 500 µg/mL as determined by Bradford Protein Assay. By using TEM, we found that MSC-sEV were bilipid-layered and nano-sized particles (Fig. 1b). Furthermore, EV surface epitopes, including CD9, CD63, and CD81, were all found on MSC-sEV by means of flow-cytometry analyses of (Fig. 1c). In addition, western blot analyses suggested that MSC-sEV were positive for specific EV markers such as CD9, CD63, CD81, Alix, and TSG101, which were all more highly expressed in MSC-sEV compared to MSCs (Fig. 1d). Moreover, the expression of negative EV marker Calnexin was detected in MSCs but not in MSC-sEV (Fig. 1d). All these results demonstrated that MSC-sEV isolated by anion exchange chromatography possessed the characteristics of exosomes.

### MSC-sEV significantly ameliorated allergic airway inflammation in mice

**Fig. 2:**
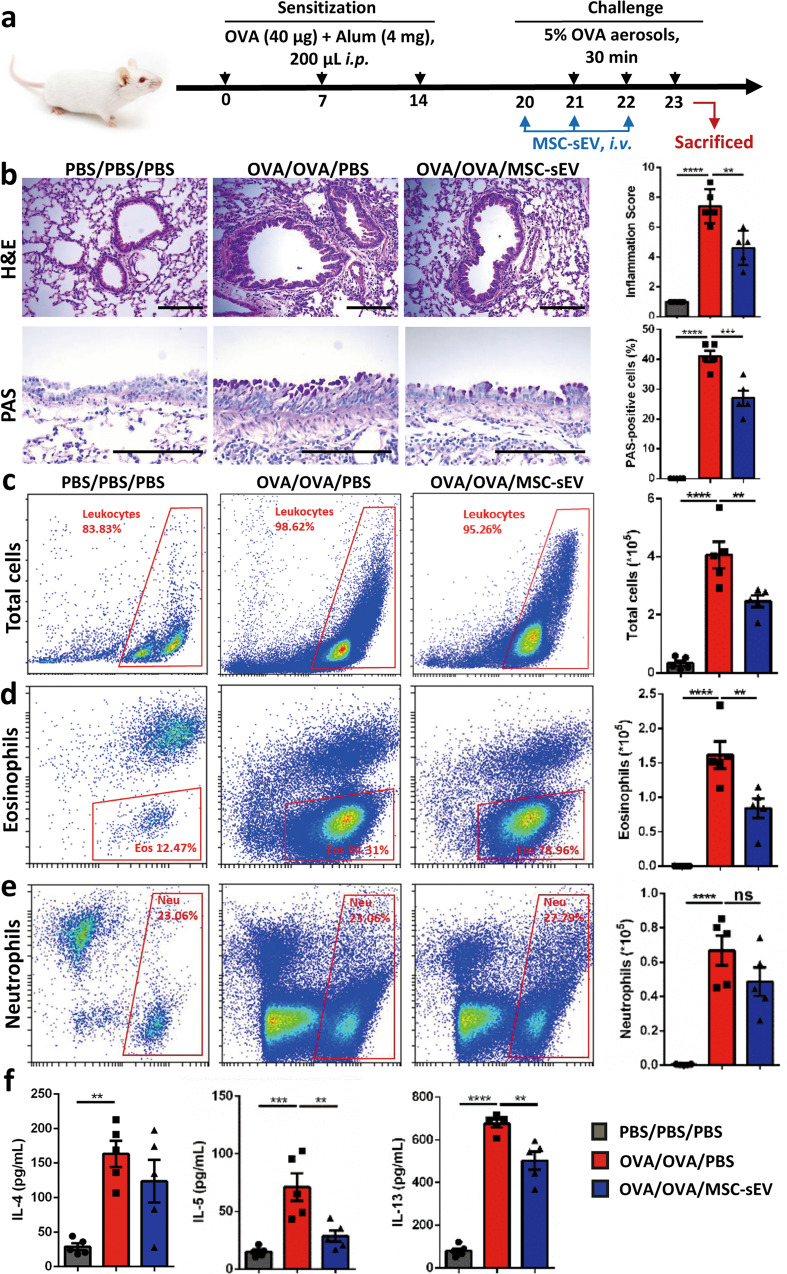
The effects of iPSC-MSC-EVs in eosinophilic allergic airway inflammation. **a** Schematic diagram for the development eosinophilic airway inflammation in mice and therapy interventions of MSC-sEV. **b** Representative figures and statistical results of H&E and PAS staining for lung tissues. **c–e** Cell counts for inflammatory cells by flow cytometry in BALF. **f** Levels of Th2-related cytokines in the BALF. **P < 0.01, ***P < 0.001, ****P < 0.0001. Alum Aluminum, BALF bronchoalveolar lavage fluids, EVs Extracellular Vesicles, H&E Hematoxylin–Eosin, i.p. intraperitoneally, MSC Mesenchymal stromal cells, ns not significant, OVA ovalbumin, PAS periodic acid-schiff, PBS phosphate buffer saline. *N* = 5 for PBS/PBS/PBS, OVA/OVA/PBS and OVA/OVA/MSC-EVs. Scale bar, 150 µm.

We firstly investigated the effects of MSC-sEV on allergic airway inflammation. The mice were sensitized with OVA and aluminum/magnesium hydroxide on days 0, 7, and 14 and challenged with 5% OVA aerosols on days 21–23 to develop eosinophilic airway inflammation (Fig. 2a). We found that systemic administration of MSC-sEV significantly reduced infiltration of inflammatory cells in peri-tracheal area, and inhibited hyperplasia of goblet cells in airway epithelium as determined by H&E and PAS staining for lung tissues, respectively (Fig. 2b). Consistently, flow-cytometry analysis of inflammatory cells in BALF suggested that numbers of total inflammatory cells and eosinophils were dramatically decreased by MSC-sEV (Fig. 2c, d). No effect was observed on the levels of neutrophils (Fig. 2e). Additionally, levels of Th2-related cytokines in BALF, including IL-4, IL-5, and IL-13, were significantly decreased or tended to be lower in those mice treated with MSC-sEV (Fig. 2f). The expression of IFN-γ and IL-17A, which were involved in neutrophilic airway inflammation, was both undetectable in BALF of all the mice (data not shown). In total, these data demonstrated that systemic administration of MSC-sEV was able to significantly prevent allergic airway inflammation in mice.

### MSC-sEV inhibited the recruitment and polarization of lung macrophages in mice with allergic airway inflammation

**Fig. 3:**
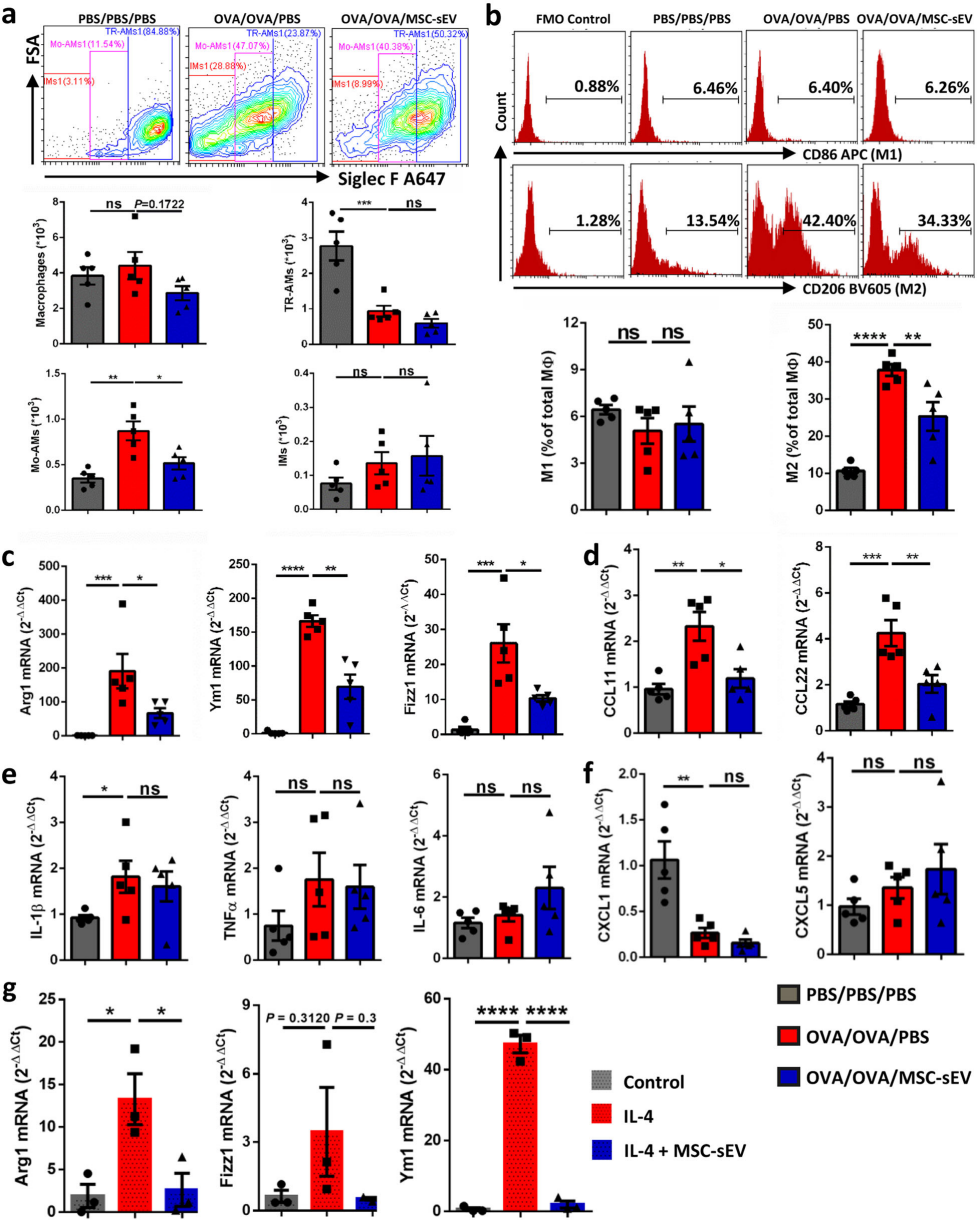
The effects of MSC-sEV on the differentiation and polarization of lung macrophages in mouse allergic airway inflammation. **a** The number of Mo-AMs, but not total macrophages, TR-AMs or IMs, was reduced by MSC-sEV. **b** Level of M2 but not M1 macrophages were decreased by MSC-sEV. **c–f** mRNA levels of M2 macrophage markers (**c**) and eosinophil-associated chemokines (**d**), M1 macrophage markers (**e**), and neutrophil-associated chemokines (**f**) were determined by RT-PCR. **g** mRNA levels of M2 macrophage markers were significantly reduced by MSC-sEV in IL-4-treated peritoneal macrophages. *P < 0.05, **P < 0.01, ***P < 0.001, and ****P < 0.0001. IMs interstitial macrophages, Mo-AMs monocytes-derived macrophages, MSCs mesenchymal stromal cells, ns not significant, OVA ovalbumin, PBS phosphate buffer saline, sEV small extracellular vesicles, TR-AMs tissue-resident macrophages.

It has been reported that pulmonary macrophages were intensively involved in the pathogenesis of asthma. However, the levels of pulmonary TR-AMs, Mo-AMs, and IMs in the pathogenesis of allergic airway inflammation remained unclear. Here, we first analyzed levels of these three macrophage subsets in different phases of allergic airway inflammation by using control, sensitized-only, and sensitized-challenged mice. Compared to the control mice, no change was observed on levels of any macrophage subsets in the sensitized-only mice. However, level of Mo-AMs was increased and TR-AMs were oppositely decreased in sensitized-challenged mice (Supplementary Fig. 3b). These results partly suggest that peripheral monocytes could be recruited to lung tissues and differentiate into Mo-AMs in allergic airway inflammation. Based on these findings, we further investigated the effects of MSC-sEV on the differentiation of Mo-AMs in the setting of eosinophilic airway inflammation. We only observed that MSC-sEV exhibited a tendency towards reducing level of total macrophages but without significant difference (Fig. 3a). Interestingly, we found that systemic administration of MSC-sEV significantly reduced level of Mo-AMs in allergic airway inflammation, and no effect was found on the proliferation of TR-AMs or IMs (Fig. 3a), suggesting that MSC-sEV were able to inhibit the differentiation of Mo-AMs in allergic airway inflammation. Additionally, we found that MSC-sEV significantly increased the levels of apoptotic macrophages (Supplementary Fig. 4), showing that MSC-sEV were able to reduce the viability of macrophages.

Furthermore, we evaluated the effects of MSC-sEV on the polarization of pulmonary macrophages in allergic airway inflammation. Flow-cytometry analysis of inflammatory cells in BALF showed that level of CD206^+^ macrophages (M2) was significantly increased in mice with allergic airway inflammation, which was abrogated by systemic administration of MSC-sEV (Fig. 3b). Consistently, no effect of MSC-sEV was found on level of CD86^+^ macrophages (M1). These data indicated that MSC-sEV were capable of reducing M2 polarization of pulmonary macrophages in eosinophilic airway inflammation. We also analyzed the expression of cytokines or chemokines that were related to polarization of macrophages. We found that systemic administration MSC-sEV dramatically downregulated the M2-related factors, such as Arg1, Ym1, and Fizz1 (Fig. 3c). Similar findings were observed for eosinophil-recruited chemokines including CCL11 and CCL22 (Fig. 3d). On the contrary, treatment of MSC-sEV displayed no effects on M1-related cytokines or neutrophil-recruited chemokines, including IL-1β, IL-6, TNF-α, CXCL1, and CXCL5 (Fig. 3e, f). In addition, we also found that MSC-sEV were able to significantly reduce the mRNA levels of Arg1 and Ym1 in IL-4 stimulated peritoneal macrophages (Fig. 3g), which further verified the inhibitory effects of MSC-sEV on the polarization of M2 macrophages.

In summary, MSC-sEV inhibited differentiation of Mo-AM and polarization of M2 macrophages in eosinophilic allergic airway inflammation.

### MSC-sEV were able to be taken in by macrophages in vivo

**Fig. 4:**
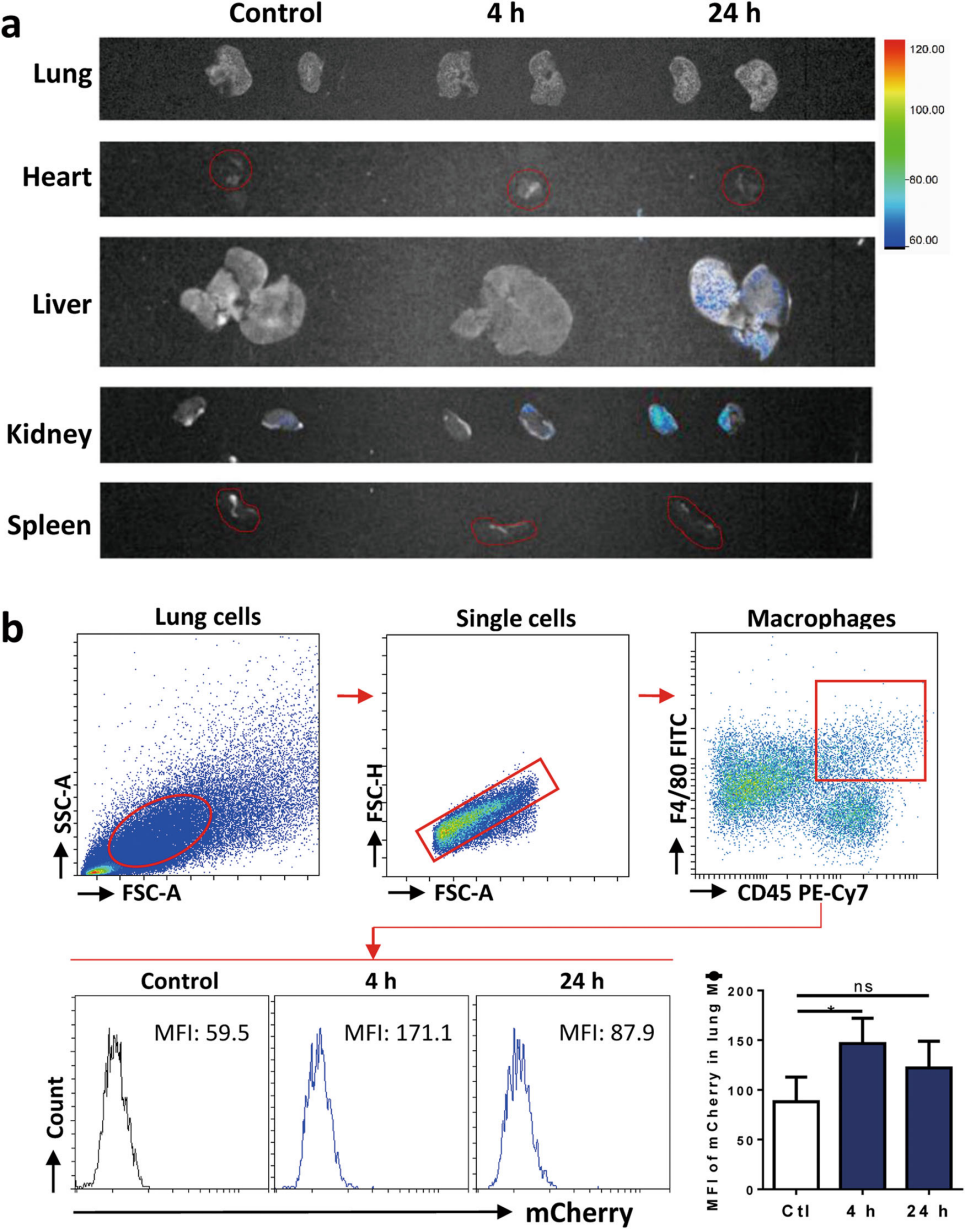
Macrophages were able to take MSC-sEV in both in vitro and in vivo. **a** Mice were administered with mCherry-labeled iPSC-MSC-sEV, and whole-body imaging was performed to detect the distribution of sEV at the indicated time points. **b** Total lung cells were isolated and MFI of mCherry in macrophages was analyzed by means of flow cytometry. Pulmonary macrophages significantly uptook MSC-EVs at 4 h post-administration. *P < 0.05. MFI mean fluorescence intensity, ns not significant.

To further elucidate the in vivo role of MSC-sEV on macrophages, we investigated the bio-distribution of MSC-sEV under the systemic delivery. Mice were injected intravenously with mCherry-labelled MSC-sEV, and the organic distribution of MSC-sEV was imaged in the lung, heart, liver, kidney, and spleen using an in vivo xtreme imaging system at 4 h and 24 h post-administration. Almost no fluorescence was detected in any organs at 4 h post-administration, and obvious fluorescence was only observed in liver and kidney at 24 h after administration (Fig. 4a). It suggests that MSC-sEV could possibly be delivered to organs via blood circulation after injection, and started to be cleared out of the body at 24 h post-administration. Additionally, we observed no fluorescence in lung, spleen or heart, which could be due to the weak fluorescent signals produced by mCherry-labelled MSC-sEV under the in vivo imaging system.

To better determine the uptake activities of pulmonary macrophages on MSC-sEV, we analyzed the mean fluorescent intensity (MFI) of mCherry in them by means of flow-cytometry analysis. We found that MFI of pulmonary macrophages was significantly increased at 4 h post-administration, respectively (Fig. 4b), suggesting that MSC-sEV could be taken in by pulmonary macrophages in vivo. It thereby provided the possibility that the function of pulmonary macrophages could be regulated by systemically-delivered MSC-sEV.

### MSC-sEV inhibited the maturation and polarization of human macrophages in vitro

**Fig. 5:**
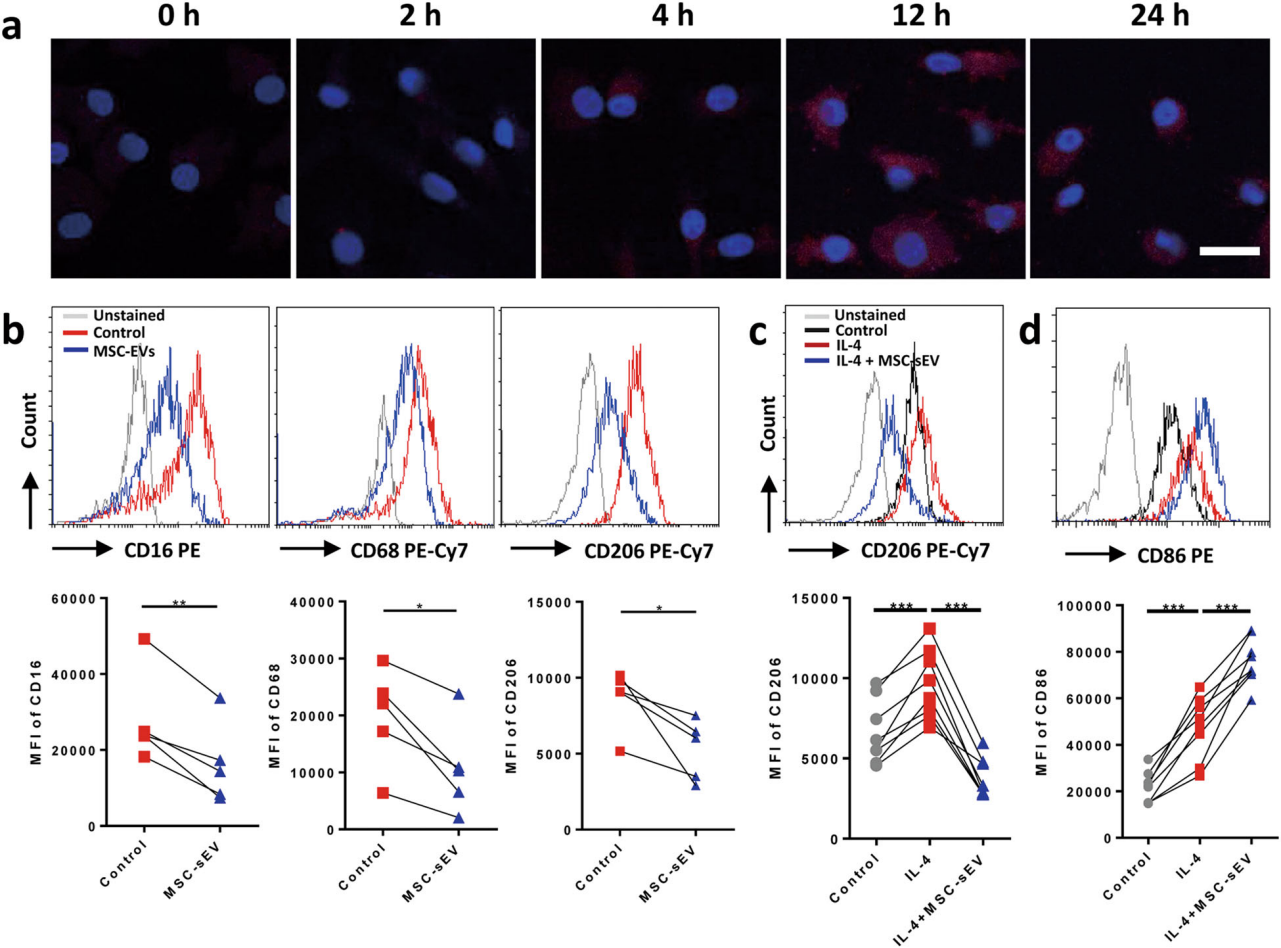
The effects of MSC-sEV on the differentiation and polarization of human Mo–Mϕ in vitro. Macrophages were induced from human CD14^+^ monocytes. **a** Macrophages were treated with mCherry-labelled MSC-sEV (red) for different times. The nuclei were counterstained with 4′,6-Diamidine-2′-phenylindole dihydrochloride (blue). **b** CD14^+^ monocytes were cultured with M-CSF for 7 days to differentiate into macrophages, and the expression of CD16, CD68 and CD206 on macrophages were determined by flow-cytometry analysis. **c**, **d** Mature macrophages were polarized into M2 and the expression of CD206 (M2 marker) and CD86 (M1 marker) was determined by flow-cytometry analysis. *P < 0.05, **P < 0.01, ***P < 0.001. MSCs mesenchymal stromal cells, MFI mean fluorescence intensity, sEV small extracellular vesicles. Scale bar, 20 µm.

We next evaluated the uptake effect of human macrophages on MSC-sEV in vitro. Human peripheral CD14^+^ monocytes were differentiated into macrophages, and incubated with mCherry-labelled MSC-sEV for different time points. We found that human macrophages were able to take MSC-sEV in a time-dependent manner, which peaked at 12 h after the treatment of MSC-sEV (Fig. 5a), which was consistent with the above results of the uptake effects of pulmonary macrophages on MSC-sEV in mice. Next, we investigated the effects of MSC-sEV on the maturation of macrophages. We found that MFI of CD16, CD68, and CD206 on the induced macrophages was significantly decreased by MSC-sEV (Fig. 5b), suggesting that MSC-sEV were able to inhibit the maturation of macrophages. This finding was also consistent with the above animal study that Mo-AMs were decreased in mice with allergic airway inflammation after the administration of MSC-sEV. We next evaluated the effects of MSC-sEV on the polarization of macrophages. We observed that MFI of CD206, the marker for M2 macrophages, was dramatically increased with the stimulation of IL-4, which was abrogated after treatment of MSC-sEV (Fig. 5c). On the contrary, MSC-sEV significantly increased expression of CD86, the marker for M1 macrophages on the IL-4-stimulated macrophages (Fig. 5d). In total, our findings demonstrated that MSC-sEV elicited strong immunoregulatory effects on the maturation and polarization of human macrophages.

### Proteomic signatures of MSC-sEV

**Fig. 6:**
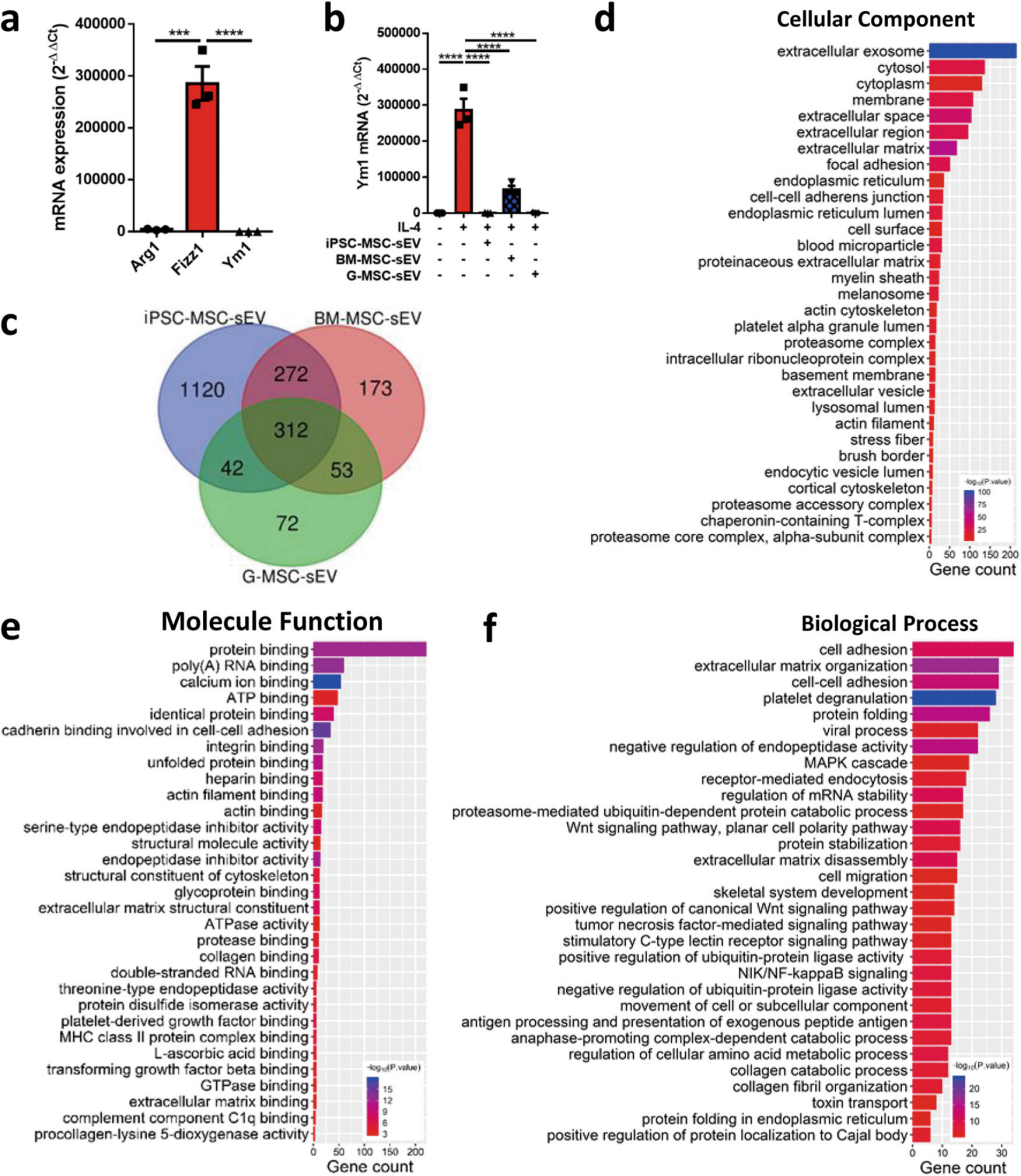
Proteomic signatures of MSC-sEV. **a**, **b** Raw 264.7 macrophages were stimulated with IL-4 for 48 h for polarization of M2 macrophages (**a**), and were treated with three types of MSC-sEV. The mRNA levels of Arg1, Fizz1, and Ym1 were determined by qRT-PCR (**b**). **c** Venn diagram for the numbers of protein identified by iPSC-MSC-sEV, BM-MSC-sEV, and G-MSC-sEV. **d–f** GO analysis of the protein co-expressed by iPSC-MSC-sEV, BM-MSC-sEV, and G-MSC-sEV. ***P < 0.001 and ****P < 0.0001. BM-MSCs bone marrow-derived mesenchymal stromal cells, G-MSCs gingiva-derived mesenchymal stromal cells, iPSC-MSCs induced pluripotent stem cell-derived mesenchymal stromal cells, sEV small extracellular vesicles.

We next examined the proteomic profiles of MSC-sEV. In order to narrow the scope of the proteins, here we prepared two more types of sEV released by BM-MSCs and G-MSCs, and an overlap about the proteins was created between these three types of sEV. Firstly, we identified that similar to iPSC-MSC-sEV, both BM-MSC-sEV and G-MSC-sEV exhibited similar characteristics in terms of diameter, structure, and specific markers (Supplementary Fig. 5a–d). Moreover, all of three types of MSC-sEV were able to reduce level of Fizz1 in IL-4 treated Raw264.7 macrophages (Fig. 6a, b), indicating that these three types of MSC-sEV exhibited similar immunoregulatory effects on polarization of M2 macrophages, and might contain common components that enabled them to display similar anti-inflammatory activities.

In total, we identified 1751, 810, and 479 proteins in sEV derived from iPSC-MSCs, BM-MSCs, and G-MSCs, respectively. Specially, 312 proteins were presented commonly in the three types of MSC-sEV (Fig. 6c). To better understand the biological function of these proteins, gene ontology enrichment analysis was conducted using DAVID 6.8^33^. In order to increase confidence levels of the analyses, we only presented the results of the 30 most relevant terms for the analyses of cellular component, molecular function, and biological process. Among the 312 shared proteins in MSC-sEV, most of the proteins were significantly involved in extracellular exosomes (68.91%), cytosol (43.9%), cytoplasm (41.67%), membrane (34.61%), and extracellular space (33.33%) in terms of cellular component (Fig. 6d, Supplementary Table 2), suggesting that MSC-sEV possessed the fundamental characteristics of exosomes. Molecular function analysis suggested that the shared proteins showed extensive binding capacities to many components, such as proteins, RNA, and calcium ion (Fig. 6e, Supplementary Table 3), which may be involved in the uptake of MSC-sEV by macrophages in our study. Additionally, these proteins were largely involved in many biological processes, such as cell adhesion, extracellular matrix organization, cell adhesion, and some signaling pathways (Fig. 6f, Supplementary Table 4).

## Discussion

In this study, we successfully isolated MSC-sEV by anion exchange chromatography and confirmed their characteristics. We then demonstrated that MSC-sEV contributed to the amelioration of allergic airway inflammation, and MSC-sEV could be taken in by macrophages and modulate the differentiation and polarization of macrophages in vitro and in vivo. Furthermore, we identified the proteomic signatures of MSC-sEV, which may be involved in their immunoregulatory function.

We and others have previously demonstrated that MSCs were effective in the therapy for allergic airway inflammation, which could be attributed to the local differentiation of MSCs, secretion of functional factors, mitochondria transfer or exosomal transfer of biological components. It has been well acknowledged that MSC-sEV possessed such advantages as high biocompatibility and low immunogenicity, and thus they have become promising cell-free therapy for some refractory diseases. In consideration of the clinical application of MSC-sEV in the future, scalable protocols must be developed for isolation of MSC-sEV. Therefore, in our study, we modified the scalable anion exchange chromatography for isolation of sEV instead of using the nonscalable differential ultracentrifugation. Moreover, we used iPSC-MSCs as cell sources in that these cells are more proliferative, less immunogenic and did not induce any tumor formation^4,30,34^, which would allow us to obtain sufficient and homogenous cells for MSC-sEV production. Collectively, we successfully developed a laboratory-grade protocol for production of MSC-sEV, which was readily to be scaled up to industry-grade.

It has been previously reported that MSC-sEV were effective in the therapy for allergic airway inflammation^14,15^ and some other lung diseases^25–27^. Consistently, we demonstrated that MSC-sEV exhibited significant therapeutic effects on allergic airway inflammation. In regards to the effects of MSC-sEV on pulmonary macrophages, only one report showed that MSC-sEV ameliorated experimental bronchopulmonary dysplasia through macrophage immunoregulation^25^, but no evidence could be found on whether and how MSC-sEV regulated pulmonary macrophages in the setting of allergic airway inflammation. It has been previously reported that pulmonary M2 macrophages were significantly increased in allergic asthma patients^20,21^. In our study we found that level of Mo-AMs was significantly increased in mice with allergic airway inflammation. In our study, we demonstrated for that MSC-sEV were able to be taken in by macrophages, and exhibited immunoregulatory effects on macrophages both in vivo and in vitro. On one hand, MSC-sEV were able to modulate the polarization of M2 macrophages in lung tissues, which were involved in the pathogenesis of allergic airway inflammation. On the other hand, MSC-sEV were capable of inhibiting the differentiation of monocytes to macrophages, which contributed to the development of allergic airway inflammation. Since MSC-sEV showed a tendency towards reducing level of total macrophages in mouse lung tissues, we cannot exclude the possibility that the decreasing trend of the total amount of macrophages could partly account for the reduced expression of M2 polarization markers in lungs of mice treated with MSC-sEV. Our data were consistent with previous study that MSC-sEV displayed modulatory effects on pulmonary macrophages phenotypes in mice with experimental bronchopulmonary dysplasia^25^. Importantly, dysfunction of alveolar macrophages is associated with the severe asthma, which is insensitive to the therapy of corticosteroid. The immunoregulatory effects of MSC-sEV on macrophages in allergic airway inflammation would provide an alternative therapeutic approach for the therapy of asthma, especially for severe asthma. In total, our data further extend our understanding of the role of macrophages in MSC-sEV mediated amelioration of allergic airway inflammation and provide preclinical evidences for the clinical application of MSC-sEV in allergic airway inflammation.

Furthermore, we analyzed the proteomic signatures of MSC-sEV in order to understand the therapeutic roles of MSC-sEV. In recent years, proteomic analyses of MSC-sEV have been reported by several studies, in which diverse MSC sources and various EV isolation methods were applied^35^. However, it has been well recognized that differences in the origins of MSCs, cell culture conditions and EV isolation methods would result in great heterogeneity of MSC-sEV. Therefore, we collected MSC-sEV-containing supernatants from iPSC-MSCs, BM-MSCs, and G-MSCs in the same way, isolated these three types of MSC-sEV with the standard protocol of anion exchange chromatography and confirmed that they all exhibited similar anti-inflammatory effects. Interestingly, we found that the three types of MSC-sEV contained different quantities of proteins and shared 312 common proteins. Our data suggested that the proteomic profiles of different types of MSC-sEV could be distinct, but these common proteins may partially account for the similar anti-inflammatory efficacies in different types of MSC-sEV. Toh et al*.* reported that MSC-sEV most probably work through a protein-based mechanism according to the comprehensive rationale of biological concentration, biochemical functionality as well as potential of timely biochemical response^36^. This led us to speculate the role of these proteins in the therapeutic function of MSC-sEV in allergic airway inflammation. We further found that MSC-sEV expressed markers of exosomes, possessed binding activities to many cell components and were involved in many biological processes, which to some extent would elucidate the molecular mechanisms for the therapeutic effects of MSC-sEV in allergic airway inflammation.

We acknowledged several limitations of our study. We only used a single dosage of MSC-sEV in our animal experiments. Further studies are required to determine the dose-dependent responses. Also, we didn’t go further to identify the specific proteins that contributed to their therapeutic effects for the reason that we conceived all the proteins acted collectively on the function of MSC-sEV. In the future, we will further investigate the potential roles of these proteins in the regulation of allergic airway inflammation.

## Conclusion

**Fig. 7:**
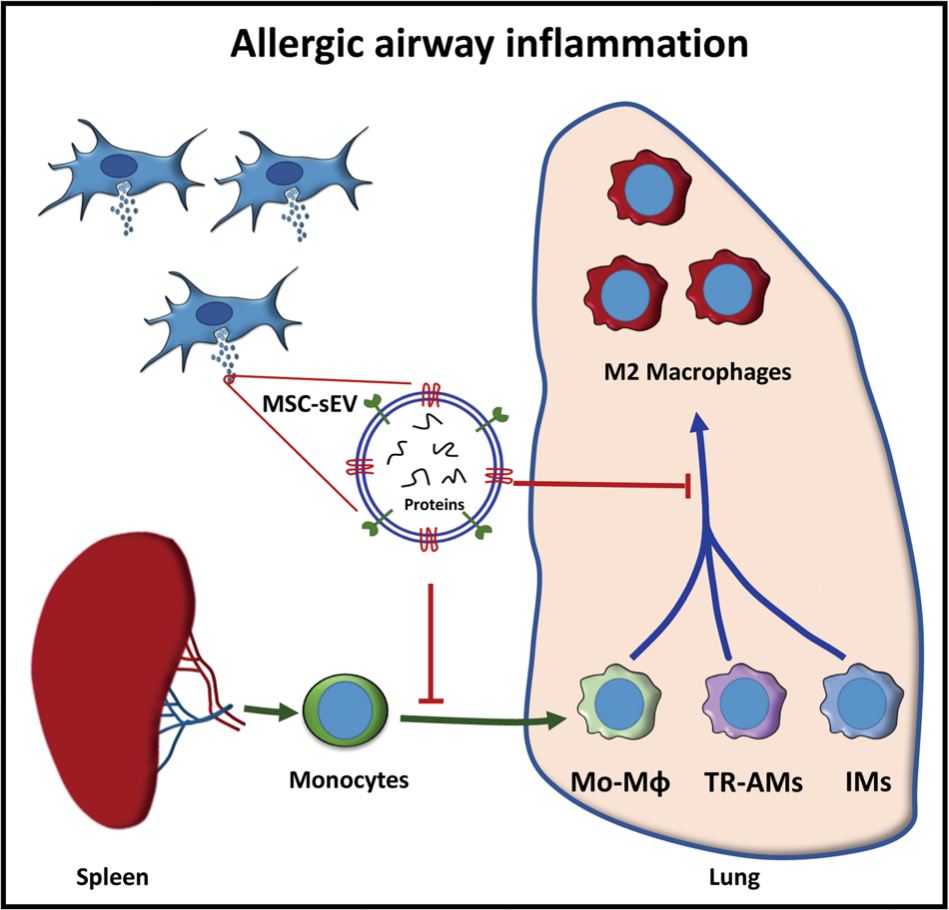
MSC-sEV ameliorated allergic airway inflammation through regulation the differentiation and polarization of pulmonary macrophages. Peripheral monocytes are recruited to lung tissues and differentiate into Mo-AMs in allergic airway inflammation. The three subsets of macrophages, including Mo-AMs, TR-AMs, and IMs, were polarized into M2 macrophages to promote the development of allergic airway inflammation. MSC-sEV display therapeutic effects in allergic airway inflammation through inhibiting the differentiation of monocytes to macrophages and polarization of M2 macrophages in lung tissues. IMs interstitial macrophages, Mo-AMs Monocytes-derived macrophages, MSCs Mesenchymal stromal cells, sEV small extracellular vesicles, TR-AMs Tissue-resident macrophages.

In summary, we successfully developed the anion exchange chromatography for the mass production of MSC-sEV. We demonstrated that MSC-sEV exhibited therapeutic effects on allergic airway inflammation through immunoregulation on pulmonary macrophages, and identified the protein profiles in MSC-sEV (Fig. 7). Our findings would provide evidences for the clinical application of MSC-sEV in allergic airway inflammation in the future.

## Supplementary information

Supplementary Figure Legends

Supplementary Fig. 1

Supplementary Fig. 2

Supplementary Fig. 3

Supplementary Fig. 4

Supplementary Fig. 5

Supplementary Table 1

Supplementary Table 2

Supplementary Table 3

Supplementary Table 4
