# Supplementary material for: Small extracellular vesicles derived from human MSCs prevent allergic airway inflammation via immunomodulation on pulmonary macrophages
Source: Cell Death Dis. 2020 Jun 1;11(6):409. doi: 10.1038/s41419-020-2606-x (PMC7264182; doi:10.1038/s41419-020-2606-x)
Supplement: Supplementary file 1 — Supplementary Figure Legends [file 41419_2020_2606_MOESM1_ESM.docx]

**Supplementary Fig. 1 Construction and characterization of mCherry-MSC-sEV.** (a) Full sequence map for pLV-mCherry:hCD63. Expression of mCherry in pLV-mCherry:hCD63-transfected iPSC-MSCs and the sEV released by the transfected iPSC-MSCs by means of microscopy (b) and flow cytometry analysis (c-d). Abbreviations: *iPSC* induced pluripotent stem cells, *MSCs* mesenchymal stromal cells, *sEV* small extracellular vesicles. Scale bar 200 μm.

**Supplementary Fig. 2 Characterization of human monocytes-derived macrophages.** (a) Flow cytometry analysis of the specific markers of macrophages; (b) White light microscopy for macrophages. Scale bar, 150 µm.

**Supplementary Fig. 3 Gating strategy for flow cytometry analyses of inflammatory cells in BALF.** (a) Cell debris (FSC-A vs. SSC-A) and doublets (FSC-A vs. FSC-H) were excluded and leukocytes (CD45^+^) were gated out. Then CD11b^-^cells were excluded from the CD45^+^ cells and Ly6G^+^ cells were identified as neutrophils. Eosinophils (Siglec F+CD64-) and macrophages (CD64^-^Siglec F) were gated on non-neutrophilic cells (Ly6G-). After exclusion of Ly-6C^+^cells, three subsets of macrophages were further identified based on the expression of Siglec F (Siglec F- for IMs, Siglec F^+^ for Mo-IMs and Siglec F^++^ for TR-AMs). (b) Percentages of the three macrophage subsets in BALF of control, OVA-sensitized and OVA-challenged mice. Abbreviations: *BALF* bronchoalveolar lavage fluid, *Eso* eosinophils, *IMs* interstitial macrophages, *Mo-AMs* monocytes-derived macrophages, *Neu* neutrophils, *OVA* ovalbumin, *TR-AMs* tissue resident alveolar macrophages.

**Supplementary Fig. 4 Effects of MSC-sEV on the viability of peritoneal macrophages.** Peritoneal macrophages were cultured in the presence or absence of MSC-sEV for 24 h and apoptotic cells were analyzed by flow cytometry analysis. ****P* < 0.001. Abbreviations: *MSCs* Mesenchymal stromal cells, *sEV* small extracellular vesicles.

**Supplementary Fig. 5 Characterization of sEV derived from BM-MSCs and G-MSCs.** (a) Nanoparticle tracking analysis of the isolated BM-MSC-sEV and G-MSC-sEV with 1:100 dilution. (b) Electron Transmission Microscopy for BM-MSC-sEV and G-MSC-sEV. (c) Western blot analyses of the exosomal specific markers. (d) Flow cytometry analyses of exosomal surface markers. Abbreviations: *BM-MSCs* bone marrow-derived mesenchymal stromal cells, *G-MSCs* gingiva-derived mesenchymal stromal cells, *sEV* small extracellular vesicles.
