# Supplementary figures and images for: Small extracellular vesicles derived from human MSCs prevent allergic airway inflammation via immunomodulation on pulmonary macrophages
Source: Cell Death Dis. 2020 Jun 1;11(6):409. doi: 10.1038/s41419-020-2606-x (PMC7264182; doi:10.1038/s41419-020-2606-x)

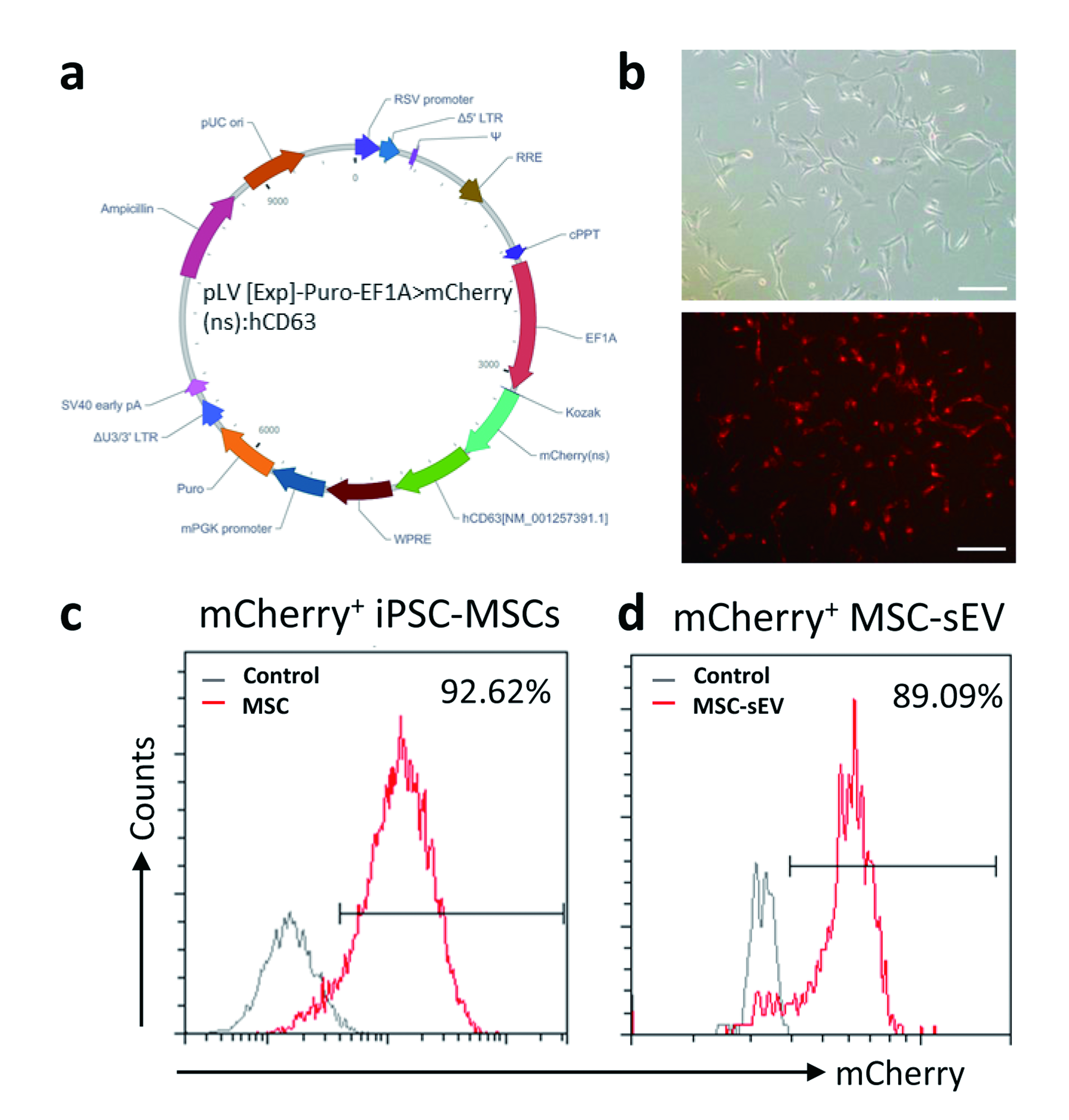

Supplement: Supplementary file 2 — Supplementary Fig. 1 [file 41419_2020_2606_MOESM2_ESM.tif]

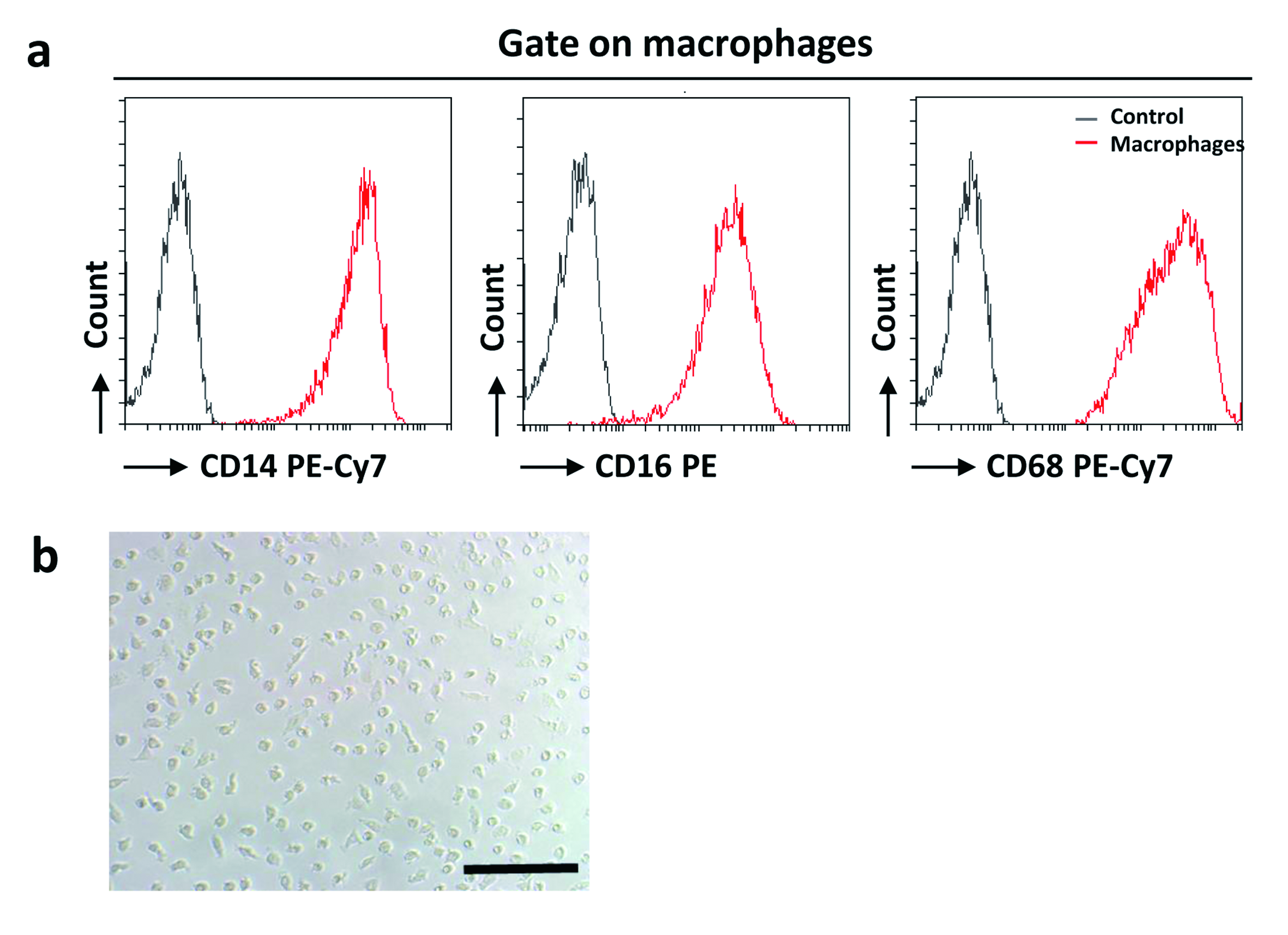

Supplement: Supplementary file 3 — Supplementary Fig. 2 [file 41419_2020_2606_MOESM3_ESM.tif]

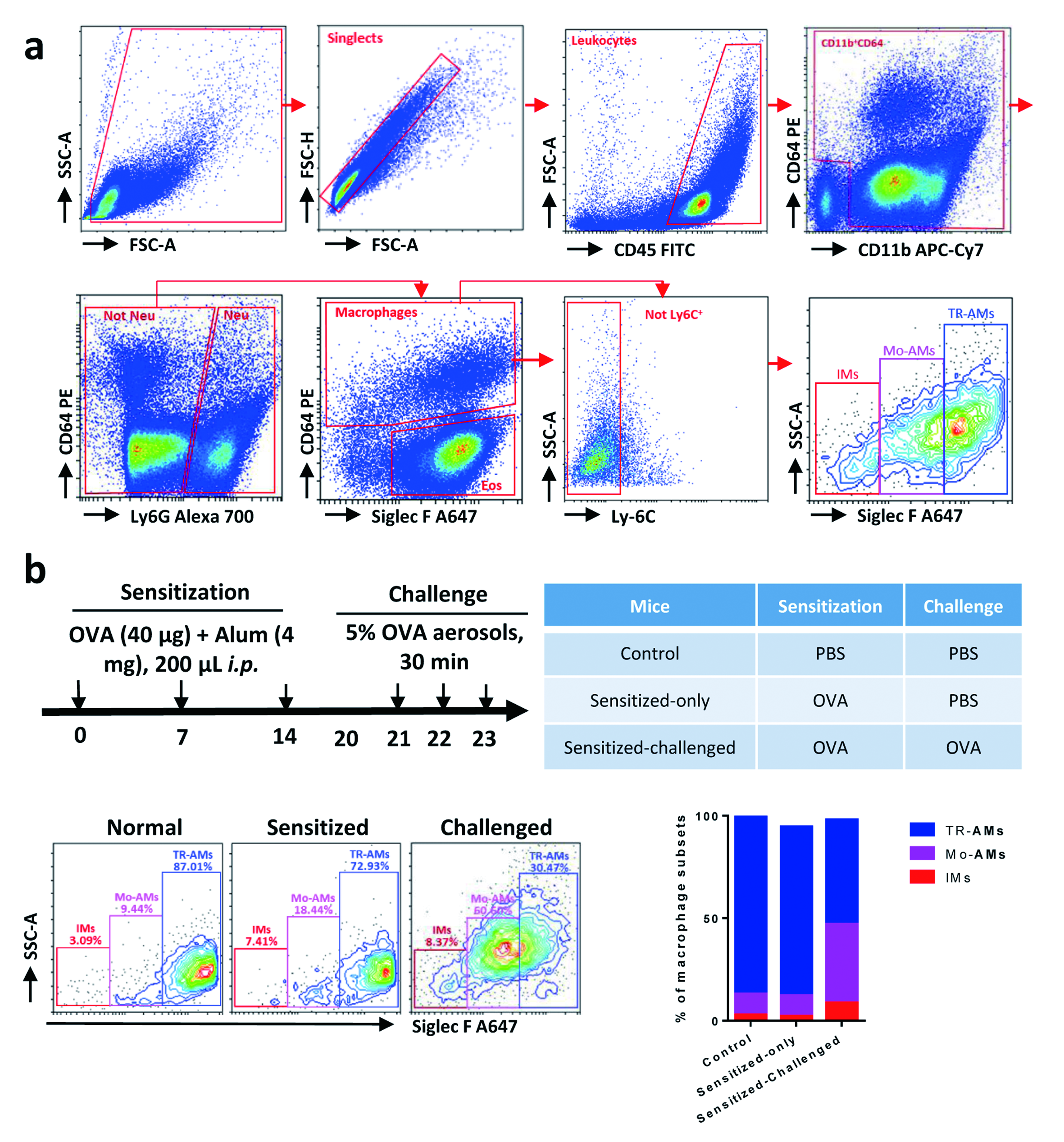

Supplement: Supplementary file 4 — Supplementary Fig. 3 [file 41419_2020_2606_MOESM4_ESM.tif]

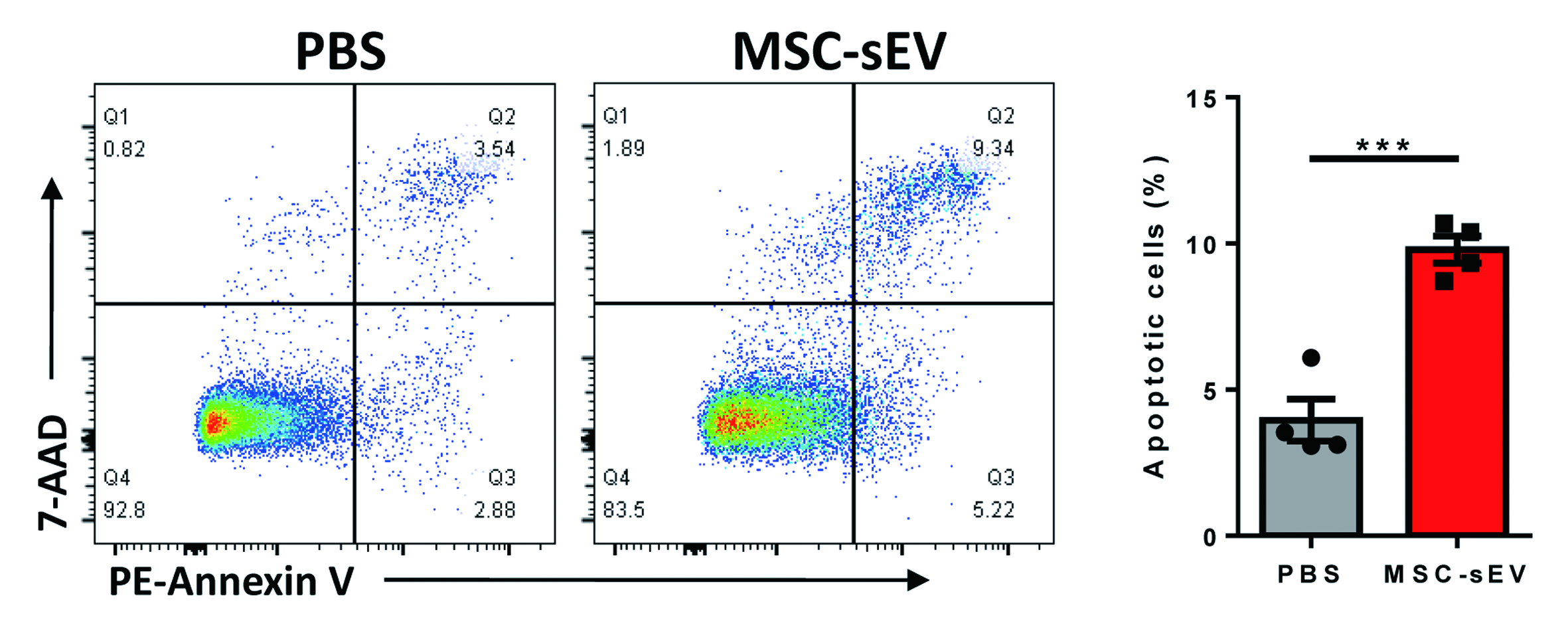

Supplement: Supplementary file 5 — Supplementary Fig. 4 [file 41419_2020_2606_MOESM5_ESM.tif]

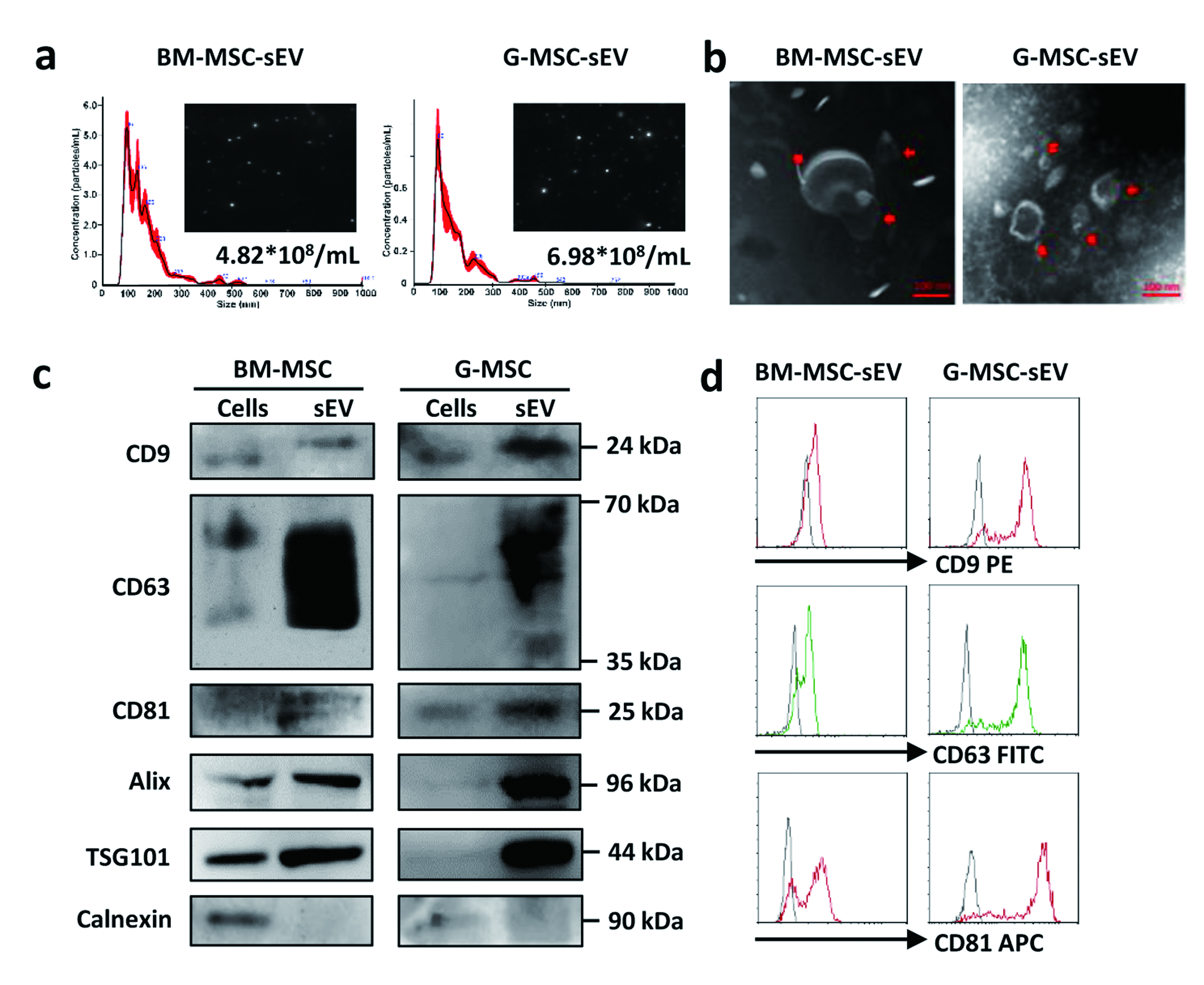

Supplement: Supplementary file 6 — Supplementary Fig. 5 [file 41419_2020_2606_MOESM6_ESM.tif]
