# Supplementary material for: Small extracellular vesicles derived from human MSCs prevent allergic airway inflammation via immunomodulation on pulmonary macrophages
Source: Cell Death Dis. 2020 Jun 1;11(6):409. doi: 10.1038/s41419-020-2606-x (PMC7264182; doi:10.1038/s41419-020-2606-x)
Supplement: Supplementary file 7 — Supplementary Table 1 [file 41419_2020_2606_MOESM7_ESM.docx]

**Supplementary Table 1** Primers for qRT-PCR.

| **Primer Name** | **Forward** | **Reverse** |
| --- | --- | --- |
| Arg 1 | CTCCAAGCCAAAGTCCTTAGAG | GGAGCTGTCATTAGGGACATCA |
| Ym1 | CAGGTCTGGCAATTCTTCTGAA | GTCTTGCTCATGTGTGTAAGTGA |
| Fizz1 | CCAATCCAGCTAACTATCCCTCC | ACCCAGTAGCAGTCATCCCA |
| CCL11 | GAATCACCAACAACAGATGCAC | ATCCTGGACCCACTTCTTCTT |
| CCL22 | AGGTCCCTATGGTGCCAATGT | CGGCAGGATTTTGAGGTCCA |
| IL-1β | GAAATGCCACCTTTTGACAGTG | TGGATGCTCTCATCAGGACAG |
| TNFα | CAGGCGGTGCCTATGTCTC | CGATCACCCCGAAGTTCAGTAG |
| IL-6 | CTGCAAGAGACTTCCATCCAG | AGTGGTATAGACAGGTCTGTTGG |
| CXCL1 | ACTGCACCCAAACCGAAGTC | TGGGGACACCTTTTAGCATCTT |
| CXCL5 | GTTCCATCTCGCCATTCATGC | GCGGCTATGACTGAGGAAGG |
| β-actin | AGAGGGAAATCGTGCGTGAC | CAATAGTGATGACCTGGCCGT |
